# The complete chloroplast genome and phylogenetic analysis of *Paris delavayi* (Melanthiaceae)

**DOI:** 10.1080/23802359.2020.1767522

**Published:** 2020-05-20

**Authors:** Li-Zhen Ling, Shu-Dong Zhang

**Affiliations:** School of Biological Science and Technology, Liupanshui Normal University, Liupanshui, China

**Keywords:** *Paris delavayi*, Melanthiaceae, chloroplast genome, phylogenetic analysis

## Abstract

*Paris delavayi* Franchet is a perennial herb of the family Melanthiaceae. In this study, the complete chloroplast (cp) genome sequence of *P. delavayi* was characterized. The cp genome is 164,195 bp in length and contains a pair of inverted repeats (33,415 bp) separated by a large (84,400 bp) and small (12,965 bp) single-copy regions. A total of 112 unique genes were predicted, including 78 protein-coding genes, 30 tRNA genes and 4 rRNA genes. The phylogenetic analysis suggested that *P. delavayi* is sister to *P. mairei* but with low support.

*Paris delavayi* Franchet is a perennial herb of the family Melanthiaceae and mainly distributed in Yunnan, Guizhou, Hubei and Hunan provinces of China (Li 1984). In Chinese Pharmacopeia, two other *Paris* plants: *P. polyphylla* var. *yunnanensis* and *P. polyphylla* var. *chinensis* are recorded as the original plants of Chonglou but become rare and endangered due to aggressive harvesting in recent decades. Many studies have reported that the major bioactive saponins have been also isolated from the rhizomes of *P. delavayi* (Liu et al. 2006; Fu et al. 2012; Liu et al. 2016; Huang et al. 2017). Therefore, *P. delavayi* could be further utilized as the potential medical resource. However, little genetic information is known about this species. To better understand and utilize this species, we sequenced and analyzed the complete chloroplast (cp) genome of *P. delavayi* using high-throughput sequencing technology.

The specimen (lpssy0305) was collected from Yushe National Forest Park (Liupanshui, Guizhou, China; 104°48′E, 26°27′N, 2,235 m) and deposited at the herbarium of the Liupanshui Normal University (LPSNU). Genomic DNA was extracted from the fresh leaves as previously described (Zhang et al. 2019) and used for the library construction and Illumina sequencing. Approximately 6 Gb raw data were used for the cp genome assembly using SPAdes (Bankevich et al. 2012). The cp genome annotation was accomplished using PGA (Qu et al. 2019).

The complete cp genome of *P. delavayi* (accession number MT038210) is 164,195 bp in length with a typical quadripartite structure containing two inverted repeats (IRs) of 33,415 bp, a large single copy (LSC) region of 84,400 bp and a small single copy (SSC) region of 12,965 bp. The overall GC content of the cp genome is 37%. A total of 112 unique genes consist of 78 protein-coding genes, 30 transfer RNA (tRNA) genes, and 4 ribosomal RNA (rRNA) genes, which is little different from other species of *Paris* (Huang et al. 2016). Among these genes, 14 genes (*atpF*, *ndhA*, *ndhB*, *petB*, *petD*, *rpl16*, *rpoC1*, *rps16*, *trnA-UGC*, *trnG-UCC*, *trnI-GAU*, *trnK-UUU*, *trnL-UAA*, and *trnV-UAC*) contain one intron and three genes (*clpP*, *rps12* and *ycf3*) have two introns.

The genus *Paris* comprises about 27 species accepted in The Plant List (http://www.theplantlist.org/) and divides into two subgenera, *Paris* and *Daiswa* (Huang et al. 2016). In recent years, several new *Paris* species, such as *P*. *lihengiana* (Xu et al. 2019), *P. tengchongensis* (Ji et al. 2017) and *P*. *nitida* (Wang et al. 2017) were reported. In this study, we constructed the phylogenetic tree and analyzed the phylogenetic position of *P. delavayi* based on the maximum likelihood (ML) and Bayesian inference (BI) methods (Ronquist et al. 2012; Stamatakis 2014). Thirteen species (*Veratrum patulum*, *V. japonicum*, *V. mengtzeanum*, *Chionographis japonica*, *Ypsilandra yunnanensis*, *Y. thibetica*, *Heloniopsis tubiflora*, *Xerophyllum tenax*, *Trillium govanianum*, *T. cuneatum*, *T. maculatum*, *T. decumbens* and *T. tschonoskii*) from other six genera of Melanthiaceae were used as the outgroups. The cp genomes of *P. delavayi* and previously published species of *Paris* were used for phylogenetic analysis. The phylogenetic tree (Figure 1) illustrates that *P. delavayi* is sister to *P. mairei* but with weak support. Therefore, the phylogenetic position of *P. delavayi* will be further studied in the future.

**Figure 1.**
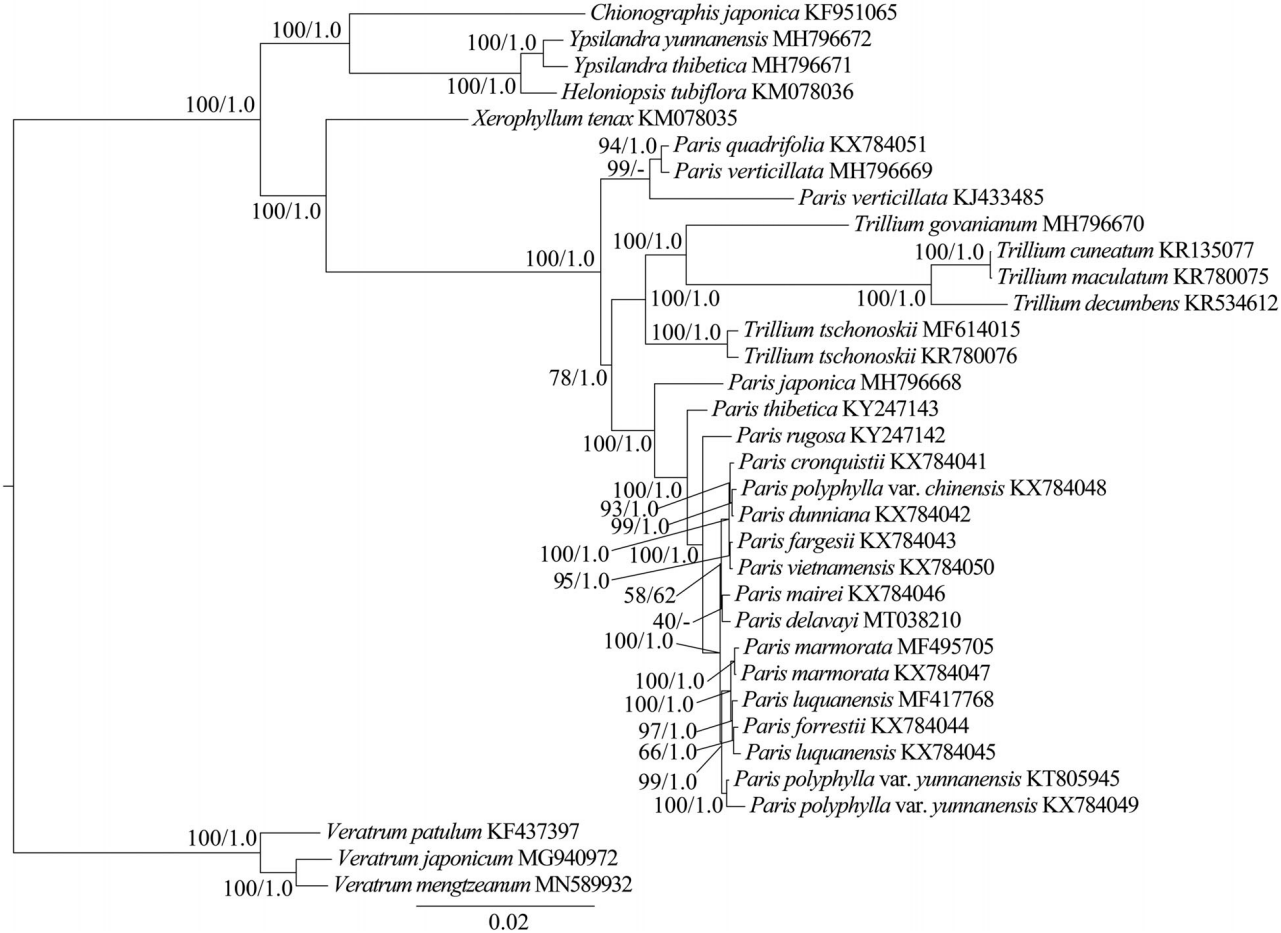
Phylogenetic tree based on whole chloroplast DNA sequences. Numbers at nodes correspond to maximum likelihood (ML) bootstrap percentages (1,000 replicates) and Bayesian inference (BI) posterior probabilities.

## Data Availability

The data that support the findings of this study are openly available in National Center for Biotechnology Information at https://www.ncbi.nlm.nih.gov/, reference number MT038210.
